# Integrin Beta 4 Protein Expression Bimodally Predicts Sensitivity to CDK4/6 Inhibition and Resistance to Immunotherapy in Breast Cancer

**DOI:** 10.1155/tbj/7668096

**Published:** 2026-04-22

**Authors:** Zhi-Min Zhu, Lei Hu, Yan-Wen Ma, Qiong-Ni Zhu

**Affiliations:** ^1^ Department of Pharmaceutics, Shanghai Eighth People’s Hospital, Shanghai, China, sh8y.com; ^2^ Department of Pharmacy, Peking University People’s Hospital, Beijing, 100044, China, pku.edu.cn; ^3^ School of Pharmaceutical Sciences, Wenzhou Medical University, Wenzhou, China, wmu.edu.cn; ^4^ Department of Pharmacy, Ruijin Hospital, Shanghai Jiao Tong University School of Medicine, Shanghai, China, shsmu.edu.cn

**Keywords:** biomarker, breast cancer, CDK4/6 inhibitor, immunotherapy resistance, ITGB4, multiomics

## Abstract

**Background:**

Integrin beta 4 (ITGB4) has been implicated in breast cancer progression, yet its clinical utility as a biomarker remains unclear due to inconsistent findings across studies. This discrepancy may stem from the failure to distinguish between RNA and protein levels.

**Methods:**

We performed an integrated multiomics analysis of ITGB4 across breast cancer subtypes using data from TCGA, CPTAC, METABRIC, and GEO cohorts. Key findings were functionally validated using CDK4/6 inhibition in luminal cells and via immunohistochemistry on triple‐negative breast cancer (TNBC) tissue microarrays.

**Results:**

ITGB4 exhibited significant RNA–protein discordance across breast cancer subtypes. High ITGB4 protein expression predicted a favorable prognosis in ER‐positive breast cancer (HR = 0.58, 95% CI: 0.39–0.86, *p* = 0.007) and enhanced sensitivity to CDK4/6 inhibitors. Conversely, high ITGB4 expression in TNBC correlated with immunotherapy resistance, characterized by elevated PD‐L1/PD‐L2 expression and reduced cytotoxic lymphocyte infiltration. Mechanistically, we identified the ESR1/miR‐342‐5p/UBE2E3 axis as a potential regulator of ITGB4 protein stability.

**Conclusion:**

ITGB4 protein expression serves as a bimodal biomarker in breast cancer, predicting CDK4/6 inhibitor sensitivity in luminal subtypes while indicating immunotherapy resistance in TNBC. ITGB4 protein thus represents a critical biomarker for guiding personalized therapy in precision oncology.

## 1. Introduction

Breast cancer remains the most commonly diagnosed malignancy among women worldwide, with substantial heterogeneity in clinical outcomes and treatment responses across molecular subtypes. The advent of CDK4/6 inhibitors has revolutionized the treatment of hormone receptor‐positive breast cancer, while immune checkpoint inhibitors have shown promise in triple‐negative breast cancer (TNBC) [1, 2]. However, reliable biomarkers to predict therapeutic response remain an unmet clinical need.

Integrin beta 4 (ITGB4), a transmembrane receptor‐mediating cell–extracellular matrix interactions, has emerged as a molecule of interest in cancer biology [3]. It can function as either an oncogene or a tumor suppressor in a tissue‐specific manner [4]. For instance, ITGB4 drives aggressiveness in cancers like pancreatic adenocarcinoma [5, 6] and lung cancer [7, 8], but its suppression can enhance chemotherapy sensitivity in IDH1‐mutant gliomas [9] and is associated with favorable prognosis in specific immune contexts [10, 11]. In TNBC, ITGB4 has been shown to promote metastasis by reprogramming cancer‐associated fibroblasts [12], while other studies highlight its potential as a target for novel immunotherapies [13, 14]. However, conflicting reports regarding its prognostic impact in breast cancer have created a paradox. A critical gap remains in understanding the utility of ITGB4 as a predictive biomarker for response to mainstream therapies, particularly CDK4/6 inhibitors and immune checkpoint blockers, which is entirely unknown. Moreover, whether discordance between its RNA and protein expression levels underlies its contradictory roles and holds clinical significance has not been systematically explored.

In this study, we performed a comprehensive multiomics analysis integrating transcriptomic, proteomic, and clinical data from multiple independent cohorts. We aimed to: (1) characterize the discordance between ITGB4 RNA and protein expression across breast cancer subtypes; (2) evaluate the prognostic and predictive value of ITGB4 protein expression; (3) investigate the relationship between ITGB4 and therapeutic response to CDK4/6 inhibitors and immunotherapy; and (4) explore the regulatory mechanisms underlying ITGB4 RNA–protein discordance. Our findings position ITGB4 protein as an actionable biomarker for personalized treatment strategies in breast cancer.

## 2. Materials and Methods

### 2.1. Study Design and Data Acquisition

This study integrated multiomics data from public repositories and validated key findings with experimental models and clinical samples. All analyses adhered to Findable, Accessible, Interoperable, and Reusable (FAIR) data principles. Data were obtained from The Cancer Genome Atlas (TCGA; https://portal.gdc.cancer.gov) for RNA‐seq, clinical, and methylation data; the Clinical Proteomic Tumor Analysis Consortium (CPTAC; https://proteomics.cancer.gov) for proteomic and phosphoproteomic profiles; the Genomics of Drug Sensitivity in Cancer (GDSC; https://www.cancerrxgene.org) for drug sensitivity data; and the Gene Expression Omnibus (GEO; https://www.ncbi.nlm.nih.gov/geo) for validation cohorts (e.g., GSE110153, GSE25066, GSE40627, GSE155605).

### 2.2. Cell Culture and Drug Treatment

MCF‐7 luminal breast cancer cells (CL‐0149, Procell) were authenticated by short tandem repeat profiling and maintained in DMEM (Gibco) supplemented with 10% fetal bovine serum (FBS) and 1% penicillin/streptomycin at 37°C under 5% CO_2_. To assess the effect of CDK4/6 inhibition, cells were treated with abemaciclib (S7158, Selleck Chemicals) at concentrations of 0.1, 1, and 5 μM for 48 h. A vehicle control (0.1% dimethyl sulfoxide) was included in all experiments.

### 2.3. Western Blot Analysis

Following treatment, cells were lysed in RIPA buffer containing protease inhibitors (Beyotime). Total protein (30 μg per lane) was separated by 8% SDS‐PAGE, transferred to PVDF membranes, and probed with primary antibodies against ITGB4 (1:1000, 21738‐1‐AP, Proteintech) and GAPDH (1:5000, ab128915, Abcam). Membranes were then incubated with HRP‐conjugated secondary antibodies (1:1000, LF102, Epizyme Biotech), and signals were detected using an ECL substrate (Epizyme Biotech). Band intensities were quantified using ImageJ software (v1.53) and normalized to GAPDH.

### 2.4. Immunohistochemistry (IHC) on Tissue Microarray

A tissue microarray (Shanghai Biochip Co., Ltd.) containing 48 TNBC specimens with annotated Ki‐67 status was used for ITGB4 protein validation, following a previously established protocol [15]. Briefly, after antigen retrieval in 10 mM sodium citrate buffer (pH 6.0) at 99°C for 22 min, sections were incubated overnight at 4°C with an anti‐ITGB4 monoclonal antibody (1:200, 21738‐1‐AP, Proteintech). Staining was visualized with DAB, and slides were scanned using a Panoramic MIDI scanner (3DHISTECH). Two independent, blinded pathologists assessed the staining intensity (scored 0–3) and the percentage of positive cells. Histochemical score (H‐score) was calculated for each sample as the product of intensity and percentage scores. H‐score was calculated as follows: H − score = (1 × percentage of weakly stained cells) + (2 × percentage of moderately stained cells) + (3 × percentage of strongly stained cells), yielding a score range of 0–300. Staining intensity was independently evaluated by two pathologists blinded to clinical outcomes. The median H‐score of the cohort was used as the threshold to stratify patients into ITGB4‐high (≥ median) and ITGB4‐low (< median) groups.

### 2.5. Bioinformatic and Statistical Analyses

Transcriptomic and Proteomic Analysis: Differential ITGB4 expression between tumor and normal tissues was analyzed using TIMER2 [16]and visualized with GEPIA2 [17]. Protein and phosphoprotein levels were interrogated via the UALCAN platform [18].

Survival Analysis: Kaplan–Meier survival curves for overall survival (OS) and disease‐free survival (DFS) were generated using GEPIA2 (median cutoff) and validated with KM‐Plotter [19] and bc‐GenExMiner v5.2 [20]. Multivariate Cox regression models, adjusted for age, stage, and subtype, were implemented using the survival package (v3.2‐10) in R.

Genetic and Epigenetic Analysis: ITGB4 copy number alterations were identified using cBioPortal [21] and GISTIC 2.0 [22]. DNA methylation data from TCGA were analyzed with SurvivalMeth [23] and MethSurv [24], and differential methylation was confirmed using DiseaseMeth [25].

Immune Microenvironment: Immune cell infiltration scores were estimated from TCGA RNA‐seq data (log2(TPM+1)) using the immunedeconv R package (v2.1.0).

Functional Enrichment and Pharmacogenomics: KEGG pathway enrichment analysis for ITGB4‐coexpressed genes and siRNA‐knockdown‐derived differentially expressed genes (GSE11506) was performed using KOBAS 3.0 [26]. Correlations between ITGB4 expression and drug IC50 values were predicted using GDSC data and the pRRophetic R package (v0.5) [27], with batch effects corrected by ComBat. Putative miRNA regulators of ITGB4 were predicted using TargetScan, miMAP, and miRWalk databases.

Stratification into ITGB4‐high and ITGB4‐low groups was based on the median expression value within each dataset for RNA‐seq analyses, or the median H‐score for IHC‐based protein analyses. The median ITGB4 expression values used for classification were 5.47 (log_2_ TPM, TCGA RNA‐seq), 0.00 (Z‐score, CPTAC proteome; KM‐Plotter, RPPA), and 170.32 (H‐score, TMA cohort).

### 2.6. Statistical Analysis

All analyses were conducted in R (v4.0.3). Group comparisons for normally distributed data used Student’s *t*‐test, while nonparametric data were analyzed with the Mann–Whitney *U* test. Survival differences were assessed by the log‐rank test. Spearman’s rank correlation coefficient was used for correlation analyses. Multiple comparisons were adjusted using the Benjamini–Hochberg false discovery rate (FDR) method. Experimental data are presented as mean ± SEM from three independent replicates. Dose–response effects were analyzed by one‐way ANOVA with Dunnett’s post hoc test.

## 3. Result

### 3.1. ITGB4 Exhibits Subtype‐Specific Expression Discordance Between RNA and Protein Levels in Breast Cancer

We commenced by delineating ITGB4 expression patterns across various cancer types. Pan‐cancer analysis revealed a tissue‐specific dichotomy: ITGB4 was suppressed in hormone‐driven malignancies (e.g., breast, ovarian) but upregulated in others (e.g., renal, pancreatic) (Figures 1(a) and 1(b)). Focusing on breast cancer, we observed a pronounced downregulation of ITGB4 mRNA in luminal subtypes compared to normal tissue (*p* = 1 × 10^−19^, Figure 1(c)), which was inversely correlated with estrogen receptor activity (*R* = −0.26, *p* = 1 × 10^−18^, Figure 1(d)). We observed that Luminal B HER2‐negative tumors exhibited lower ITGB4 expression compared to Luminal A, which reflecting their higher proliferative activity index. This RNA–protein discordance was validated in an independent TNBC tissue microarray, where ITGB4 protein was significantly overexpressed in tumors compared to adjacent normal tissue (H‐score: 106.99 vs. 97.55; *p* = 0.017, Figures 1(e) and 1(g)). Furthermore, high ITGB4 protein expression strongly correlated with enhanced tumor–stromal connectivity (*R* = 0.683, *p* < 0.001, Figure 1(f)). We also found a significant association between high ITGB4 expression and elevated tumor proliferation, with 93.3% (28/30) of ITGB4‐high tumors exhibiting a Ki‐67 index ≥ 30%, compared to 80.0% (12/15) in the ITGB4‐low group (*p* = 0.045, Table 1). Strikingly, proteomic analysis revealed a discordant pattern: While luminal tumors maintained low ITGB4 protein, TNBC exhibited significant partial recovery of ITGB4 protein levels (*p* < 0.05 vs. Luminal; Figure 1(h)). ITGB4 expression exhibited subtype‐ and stage‐dependent patterns. Advanced‐stage tumors (Stage II/III) displayed progressive protein level decline compared to normal controls (*p* < 0.01; Figures 1(i) and 1(j)). This pattern suggests a link between ITGB4 expression dynamics and aggressive tumor characteristics. These results establish a fundamental discrepancy between ITGB4 transcription and translation in breast cancer, particularly in TNBC, where elevated protein is linked to stromal remodeling.

FIGURE 1ITGB4 exhibits subtype‐specific expression discordance between RNA and protein levels in breast cancer. (a) ITGB4 mRNA expression across 33 cancer types from TCGA. Tumor tissues (red) are compared to normal tissues (blue). Key cancer types are labeled. Significance: ^∗∗∗^ *p* < 0.001, ^∗∗^ *p* < 0.01 (two‐tailed *t*‐test). (b) ITGB4 protein expression across 10 cancer types from CPTAC. Tumor tissues (red) are compared to normal tissues (blue). Significance: ^∗∗∗^ *p* < 0.001, ^∗∗^ *p* < 0.01 (two‐tailed *t*‐test). (c) ITGB4 mRNA expression levels across breast cancer subtypes and normal breast tissue in the TCGA cohort (*p* = 1 × 10^−19^, ANOVA). Sample sizes: normal (*n* = 572), Luminal A (*n* = 421), Luminal B (*n* = 194), HER2‐enriched (*n* = 67), basal‐like (*n* = 140). (d). Scatter plot illustrating the inverse correlation between ITGB4 mRNA levels and ESR1 expression, indicating potential suppression by estrogen receptor signaling (Spearman’s *R* = −0.26, *p* = 1 × 10^−18^). (e) H‐scores from immunohistochemical (IHC) staining of ITGB4 in TNBC tumors versus paired adjacent normal tissues (*n* = 48 pairs; *p* = 0.017). (f) Correlation between ITGB4 H‐score and a tumor–stromal connectivity signature (*R* = 0.683, *p* < 0.001). (g) Representative IHC images (40x magnification) showing ITGB4 staining (brown) in a TNBC tumor and adjacent normal tissue. Scale bar: 50 µm (*n* = 48 pairs; *p* = 0.017). (h) Expression levels of ITGB4 in tumor samples from patients achieving pathological complete response (pCR; blue) versus those with residual disease (RD; red) following neoadjuvant therapy. Data are presented as mean ± SEM. ^∗∗∗^ *p* < 0.001. (i) ITGB4 protein levels across breast cancer subtypes and normal tissue from CPTAC proteomic data. Significance: luminal vs. normal, ^∗∗∗^ *p* < 0.001; luminal vs. TNBC, ^∗^ *p* < 0.05. Boxplots in (c, d, h) represent median ± IQR. Luminal A *n* = 18; Luminal B *n* = 64; HER2+ *n* = 10, TNBC *n* = 16. (j) ITGB4 protein expression levels stratified by pathological tumor stage (normal, Stage I, Stage II, Stage III) in the CPTAC cohort. Advanced‐stage tumors show a progressive decline compared to normal tissues (^∗∗^ *p* < 0.01).

**Figure:**
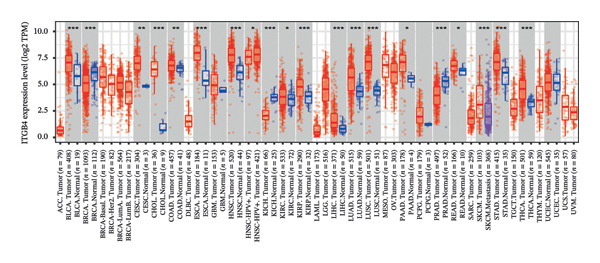
(a)

**Figure:**
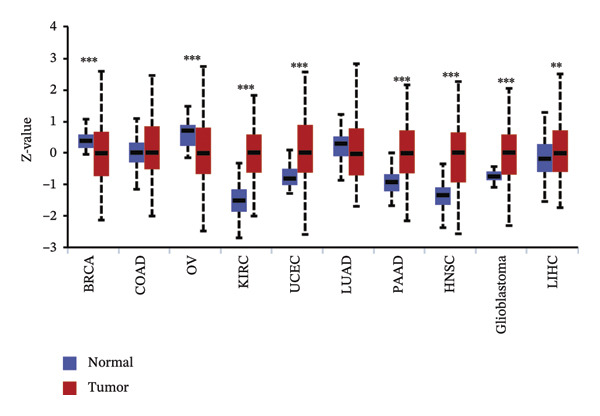
(b)

**Figure:**
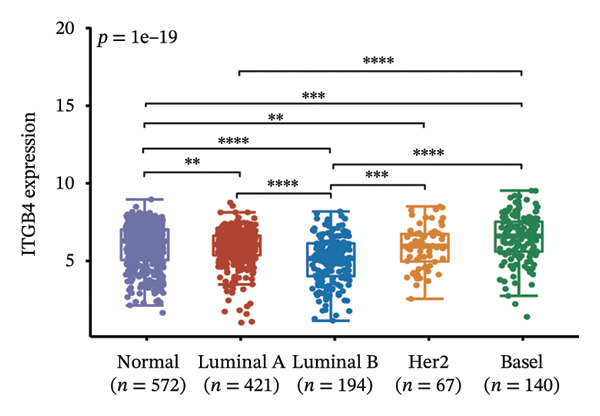
(c)

**Figure:**
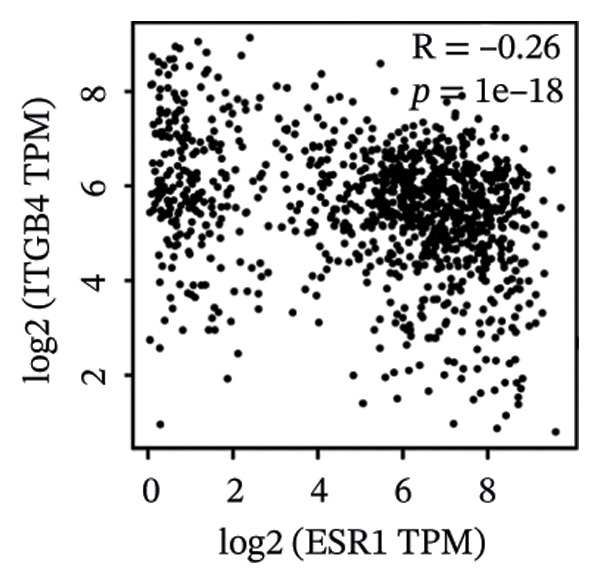
(d)

**Figure:**
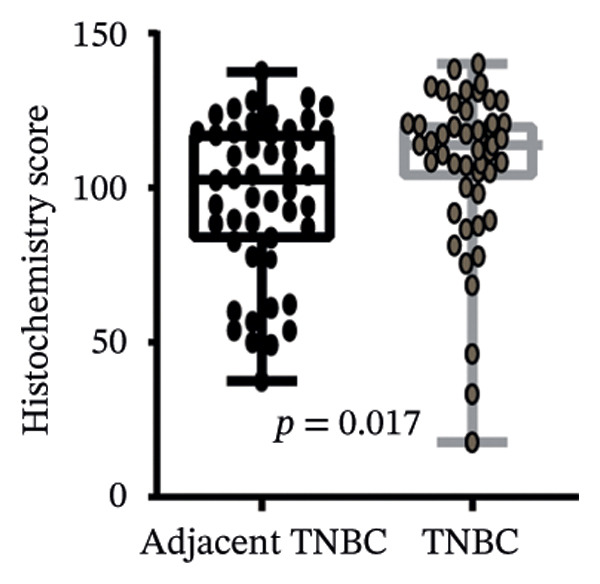
(e)

**Figure:**
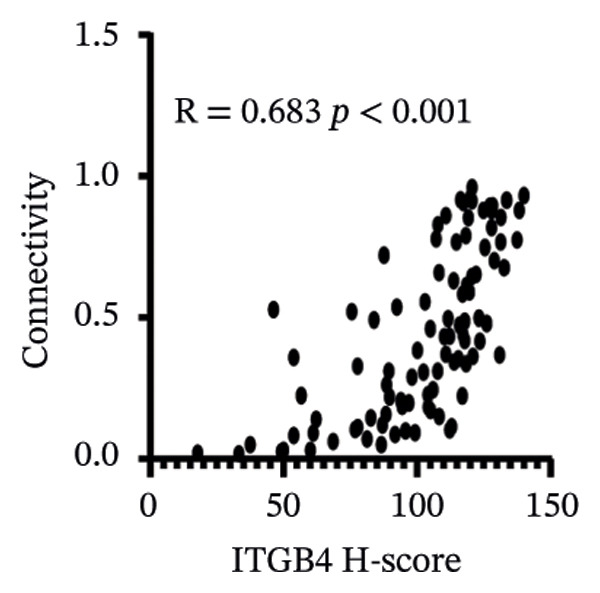
(f)

**Figure:**
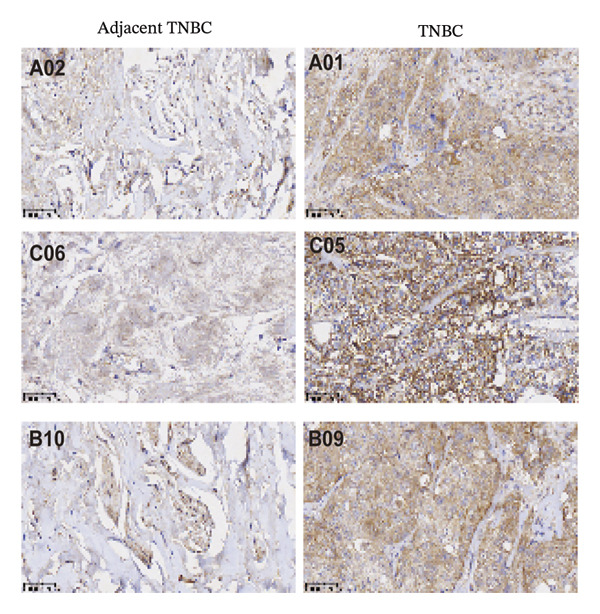
(g)

**Figure:**
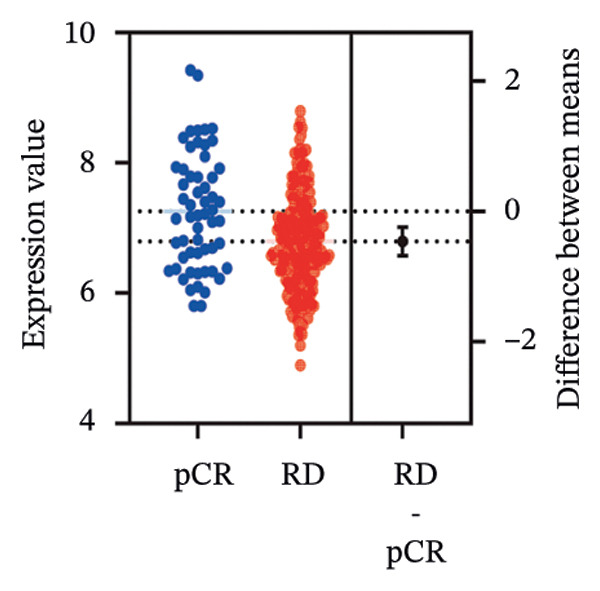
(h)

**Figure:**
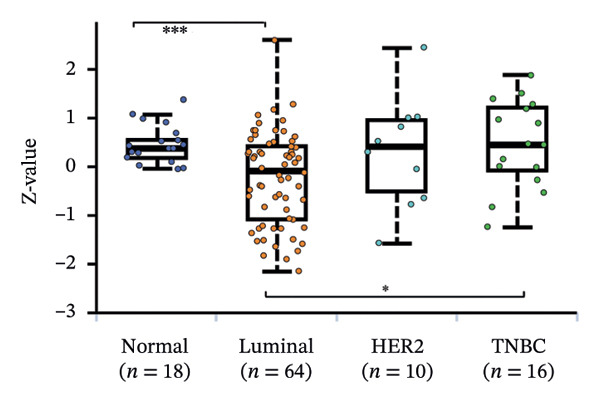
(i)

**Figure:**
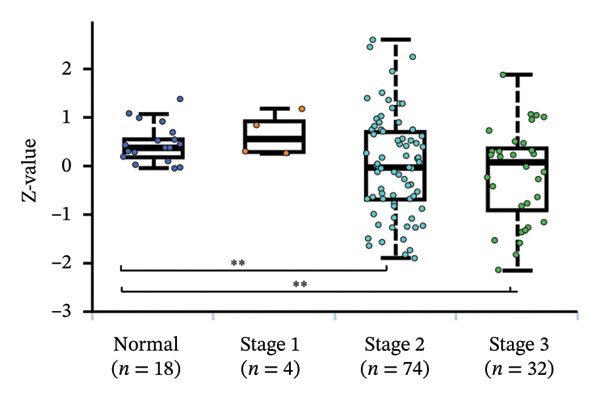
(j)

**TABLE 1:** Association between ITGB4 H‐score and clinical characteristics.

Characteristics	N	High	Low	*p*
Site				0.551
Right	19	14	5	
Left	29	19	10	
Age				0.369
≤ 65	36	26	10	
> 65	12	7	5	
T				0.775
T1	19	12	7	
T2	26	19	7	
T3	3	2	1	
N				0.810
N0	30	22	8	
N1	6	4	2	
N2	4	2	2	
N3	3	2	1	
NX	5	3	2	
M0	48	33	15	
KI67				0.045
< 30	5	2	3	
≥ 30	43	28	12	

### 3.2. High ITGB4 Protein Expression Predicts Favorable Prognosis in ER‐Positive Breast Cancer

The expression discordance prompted us to investigate the prognostic impact of ITGB4 at both RNA and protein levels. Survival analysis revealed a striking paradox. Elevated ITGB4 RNA expression was associated with a trend toward worse OS (HR = 1.42, *p* = 0.073, Figure 2(a)) and significantly poorer progression‐free survival (PFS; HR = 1.32, *p* = 0.026, Figure 2(d)). In stark contrast, high ITGB4 protein expression was a robust predictor of improved clinical outcomes. Patients with high ITGB4 protein had significantly longer OS (ER‐negative: HR = 0.35, *p* = 0.023, Figure 2(b), ER‐positive: HR = 0.22, *p* = 0.0041, Figure 2(c)). A consistent protective effect of high ITGB4 protein was also observed for PFS in ER‐negative patients (HR = 0.45, *p* = 0.0045, Figure 2(e)) and RFS in ER‐positive patients (HR = 0.61, *p* = 0.016, Figure 2(f)). Thus, the prognostic value of ITGB4 is entirely dependent on its molecular context, with protein expression serving as a favorable biomarker, independent of its RNA‐level associations.

FIGURE 2Prognostic impact of ITGB4 is dictated by its expression level (RNA vs. protein). (a) Kaplan–Meier curve for overall survival (OS) stratified by ITGB4 RNA expression in (KM‐Plotter cohort). (b, c) Kaplan–Meier curves for overall survival (OS) stratified by ITGB4 protein expression in two independent cohorts (Liu et al., *n* = 124, ER‐negative, PR‐negative, with all patient’s lymph node‐negative. Tang et al., *n* = 65, subset analyzed: ER‐positive cases only (*n* = 32); within this subset, 15 patients were lymph node‐positive and 17 were lymph node‐negative. (d) Kaplan–Meier curve for progression‐free survival (PFS) stratified by ITGB4 RNA expression. (e) Kaplan–Meier curve for PFS stratified by ITGB4 protein expression (Liu et al., ER‐negative *n* = 124). (f) Kaplan–Meier curve for RFS stratified by ITGB4 protein expression (Demarchi et al., *n* = 112, ER‐positive; 43 patients were lymph node‐positive and 61 were lymph node‐negative). In all Kaplan–Meier curves, red lines indicate the high‐expression group, and blue lines indicate the low‐expression group.

**Figure:**
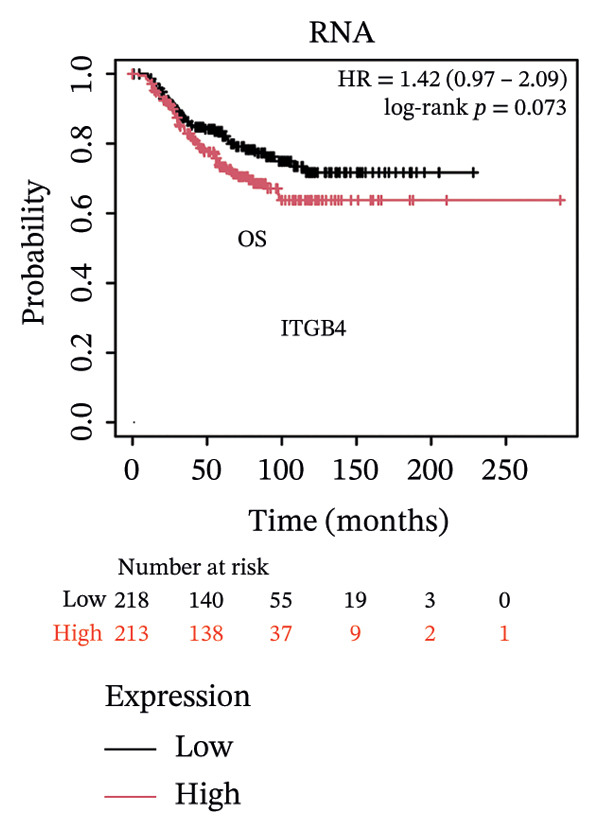
(a)

**Figure:**
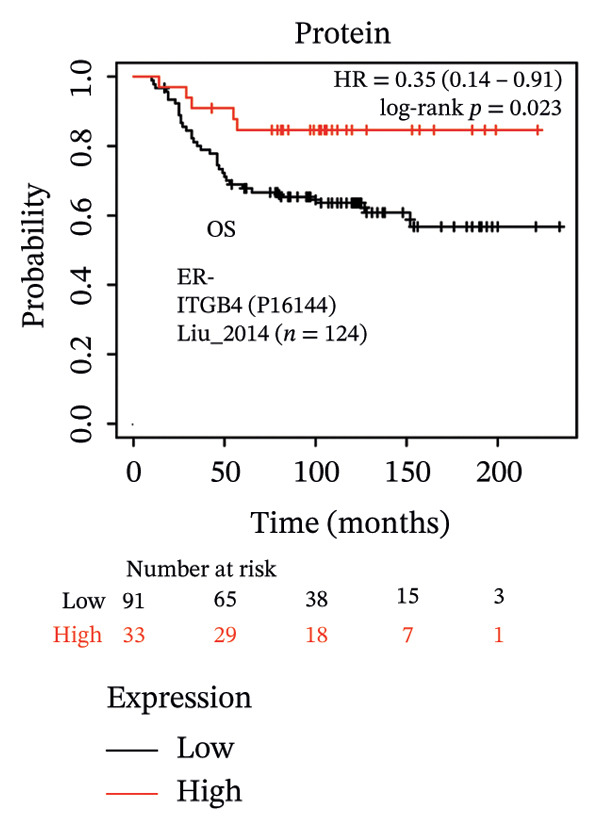
(b)

**Figure:**
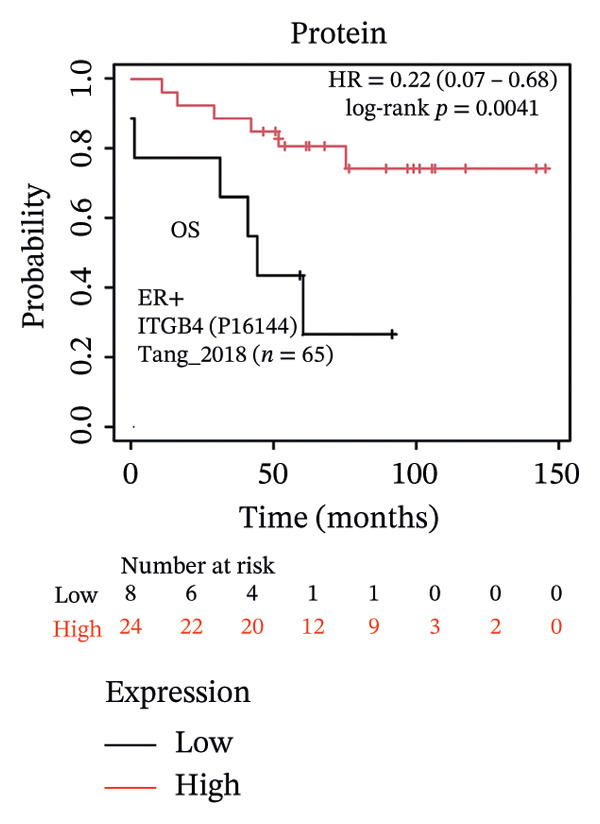
(c)

**Figure:**
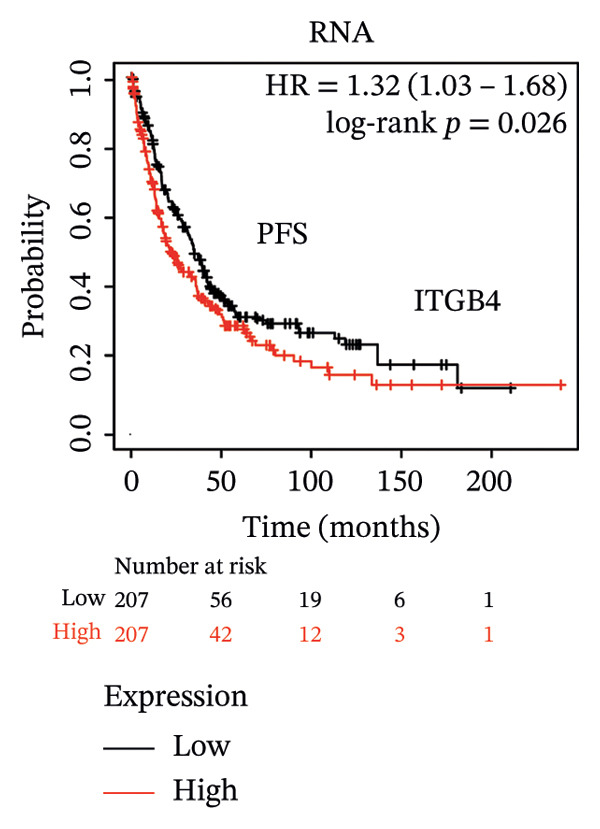
(d)

**Figure:**
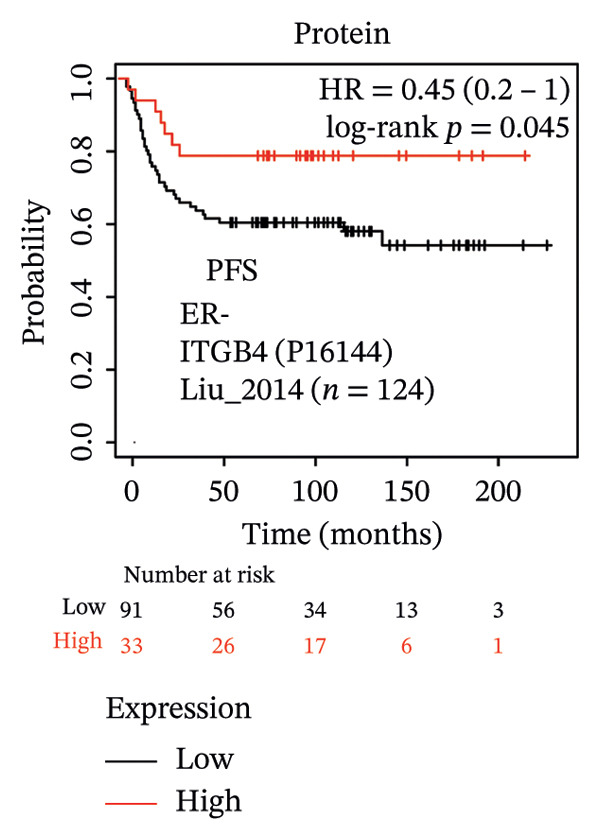
(e)

**Figure:**
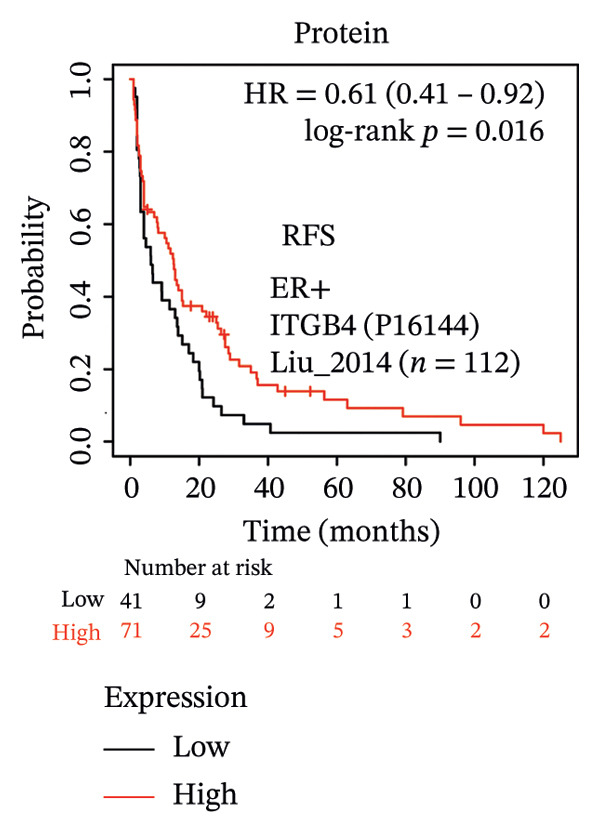
(f)

### 3.3. ITGB4 Protein Expression Predicts Sensitivity to CDK4/6 Inhibitors

We next assessed the predictive value of ITGB4 for therapy response. Pan‐cancer pharmacogenomic analysis identified a significant negative correlation between ITGB4 expression and resistance to CDK4/6 inhibitors, including palbociclib (Luminal BRCA: *R* = −0.26, *p* = 2.39 × 10^−11^, Figures 3(a) and 3(b)). Functional validation in luminal MCF‐7 cells confirmed that treatment with the CDK4/6 inhibitor abemaciclib led to a dose‐dependent suppression of ITGB4 protein, with a 69.4% reduction at 5 μM (*p* < 0.01, Figures 3(c) and 3(d)), suggesting that ITGB4 loss may increase dependency on cyclin D‐CDK4/6 signaling. Conversely, ITGB4 expression predicted resistance to chemotherapy. In breast cancer xenograft models, docetaxel‐resistant tumors exhibited higher ITGB4 levels than their sensitive counterparts (*p* = 0.0129, Figure 3(e)). Consistently, in a neoadjuvant chemotherapy cohort, patients with high ITGB4 had a significantly worse prognosis (HR = 2.01, *p* = 0.032, Figure 3(f)), indicating resistance. This positions the ITGB4 protein as a dual biomarker, predicting both sensitivity to CDK4/6 inhibition and resistance to taxane‐based chemotherapy.

FIGURE 3ITGB4 expression predicts response to CDK4/6 inhibition and chemotherapy. (a) Heatmap of Spearman correlation coefficients between ITGB4 expression and drug IC50 values across cancer types (GDSC/TCGA data). Blue indicates sensitivity (negative correlation), and red indicates resistance. ^∗^ *p* < 0.05. (b) Correlation between ITGB4 expression and sensitivity to palbociclib (a CDK4/6 inhibitor) in luminal breast cancer (BRCA) (*R* = −0.26, *p* = 2.39 × 10^–11^). (c) Dose‐dependent suppression of ITGB4 protein levels in MCF‐7 cells treated with the CDK4/6 inhibitor abemaciclib for 48 h. Data are mean ± SEM (*n* = 3 independent experiments). ^∗∗^ *p* < 0.01 vs. DMSO control (one‐way ANOVA with Dunnett’s test). (d) Representative Western blot showing ITGB4 and GAPDH (loading control) expression following abemaciclib treatment (*n* = 3 independent experiments). (e) ITGB4 expression levels in docetaxel‐sensitive versus docetaxel‐resistant xenograft models (GSE110153 dataset; ^∗^ *p* = 0.0129, *t*‐test). (f) Kaplan–Meier curves for survival in patients receiving neoadjuvant chemotherapy (HR = 2.01, *p* = 0.032) stratified by ITGB4 expression.

**Figure:**
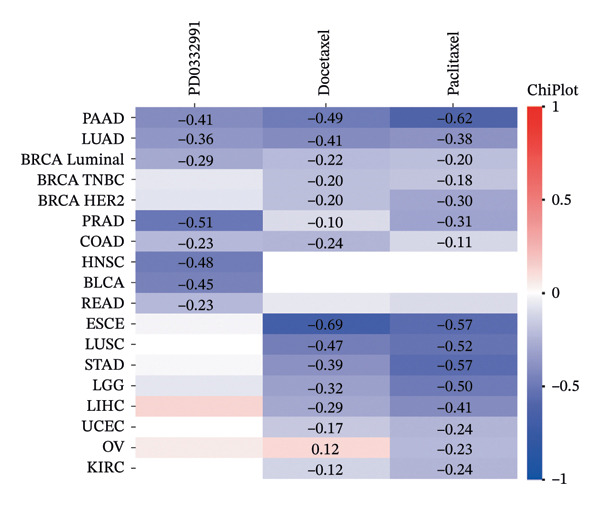
(a)

**Figure:**
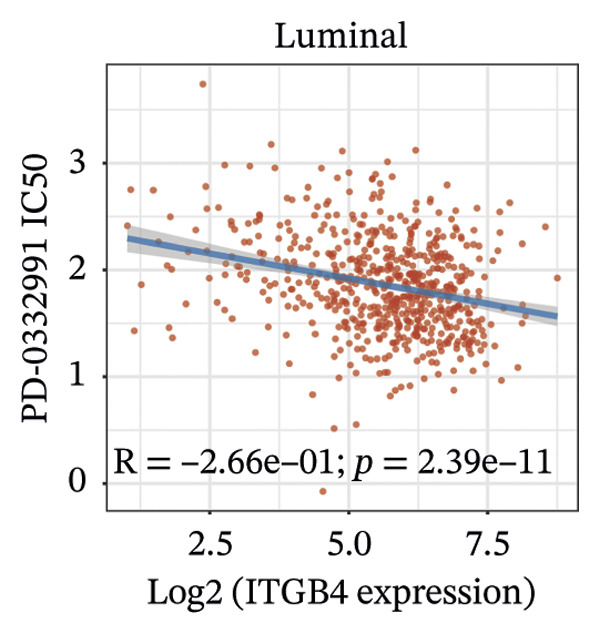
(b)

**Figure:**
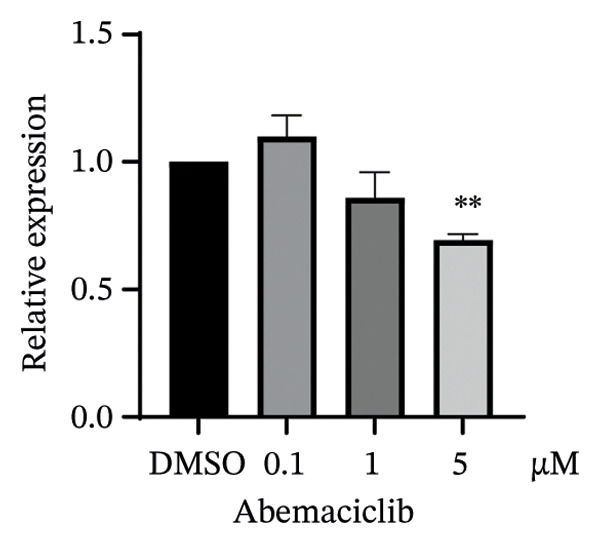
(c)

**Figure:**
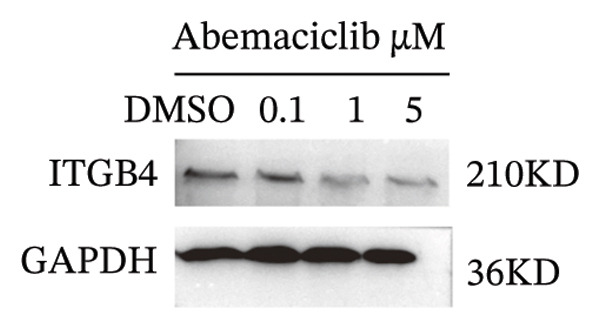
(d)

**Figure:**
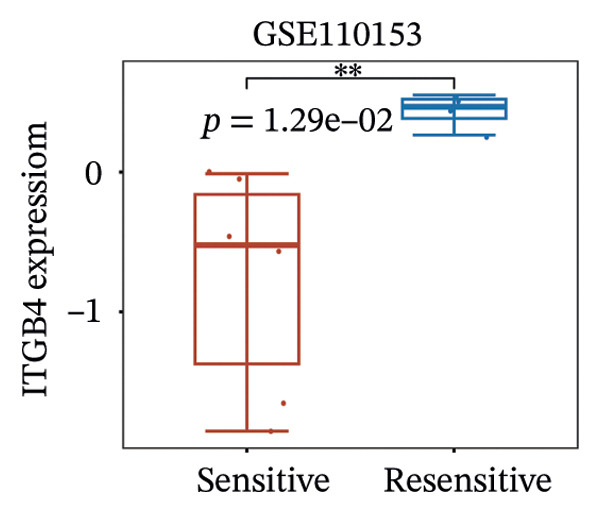
(e)

**Figure:**
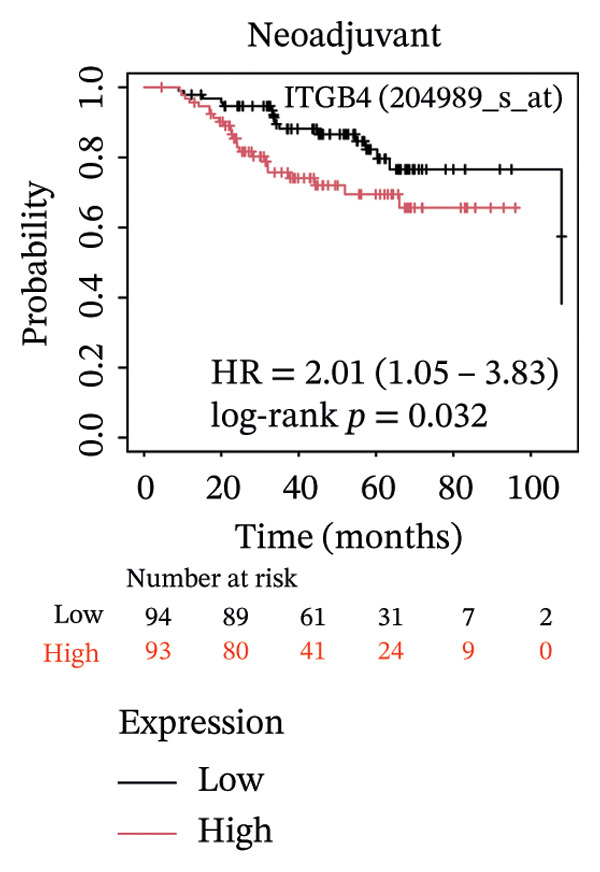
(f)

### 3.4. ITGB4 Expression Associates With Immunotherapy Resistance in TNBC

Considering the distinct biological behavior of ITGB4 across subtypes, we investigated its relationship with immunotherapy response markers. Comparative analysis revealed that ITGB4‐high tumors exhibited significantly altered immune cell infiltration compared to ITGB4‐low tumors, characterized by reduced levels of CD4+ T cells (*p* < 0.05) and increased infiltration of myeloid dendritic cells and neutrophils (*p* < 0.001 for both) (Figures 4(a) and 4(b)). Furthermore, evaluation of immune checkpoint molecules showed that ITGB4‐high breast cancers expressed significantly higher levels of PD‐L1 (CD274, *r* = 0.52, *p* < 0.001) and PD‐L2 (PDCD1LG2, *r* = 0.48, *p* < 0.001) (Figure 4(c)), suggesting a distinct immune evasion mechanism.

FIGURE 4ITGB4 expression modulates the immune microenvironment and predicts resistance to immune checkpoint inhibitors. (a) Differences in the abundance of tumor‐infiltrating immune cells between ITGB4‐high and ITGB4‐low breast cancer samples. Key significantly altered cell types are labeled. Significance: ^∗^ *p* < 0.05, ^∗∗∗^ *p* < 0.001. (b) The relative percentage abundance of various tumor‐infiltrating immune cells in each sample from the cohort. The bar plot shows the composition for individual samples (*x*‐axis), with different colors representing distinct immune cell types. (c) Differential mRNA expression of eight key immune checkpoint genes between ITGB4‐high (red) and ITGB4‐low (blue) groups in the TCGA BRCA cohort. CD274 (PD‐L1) and PDCD1LG2 (PD‐L2) are significantly upregulated, while LAG3 is downregulated in the ITGB4‐high group (^∗^ *p* < 0.05). (d, e) Kaplan–Meier survival analysis for patients treated with PD‐1 inhibitors. (d) Overall survival (OS). (e) Progression‐free survival (PFS). Hazard ratios (HRs), *p* values, and median survival times are indicated. (f, g) Kaplan–Meier survival analysis for patients treated with PD‐L1 inhibitors. (f) OS. (g) PFS. (h, i) Kaplan–Meier survival analysis for patients treated with CTLA‐4 inhibitors. (h) OS. (i) PFS. For all survival curves (d–i), data were analyzed using the log‐rank test. HR and *p* values are shown.

**Figure:**
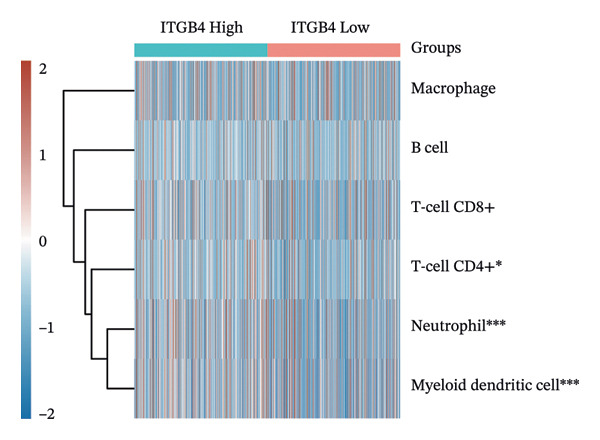
(a)

**Figure:**
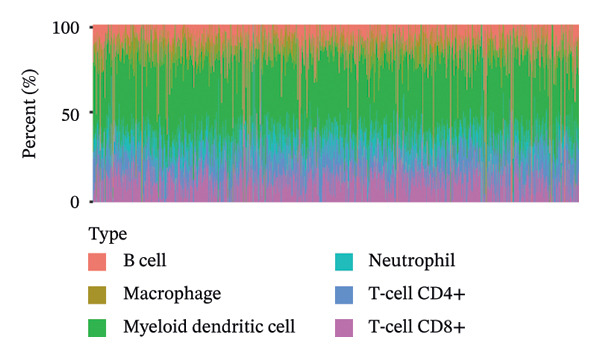
(b)

**Figure:**
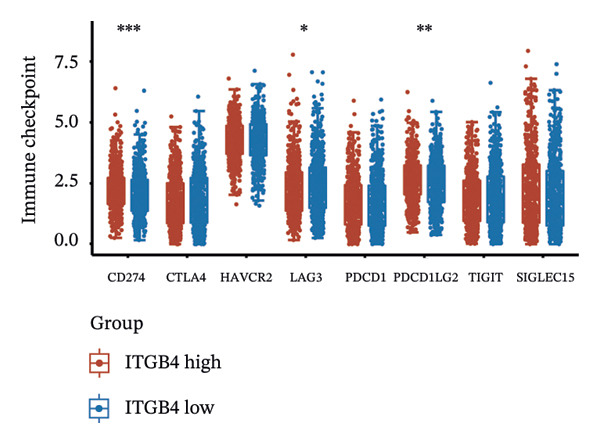
(c)

**Figure:**
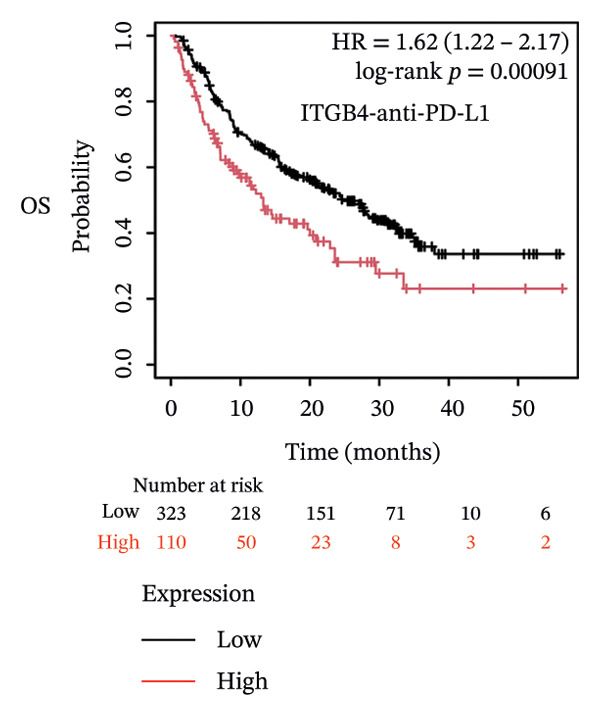
(d)

**Figure:**
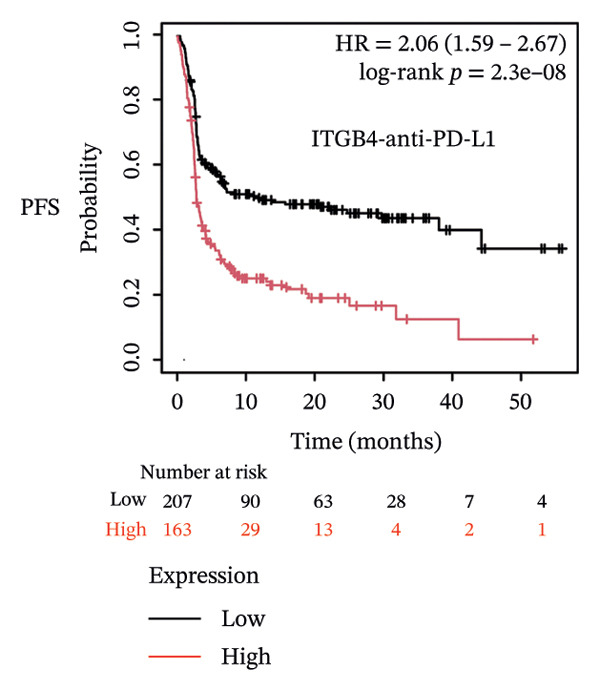
(e)

**Figure:**
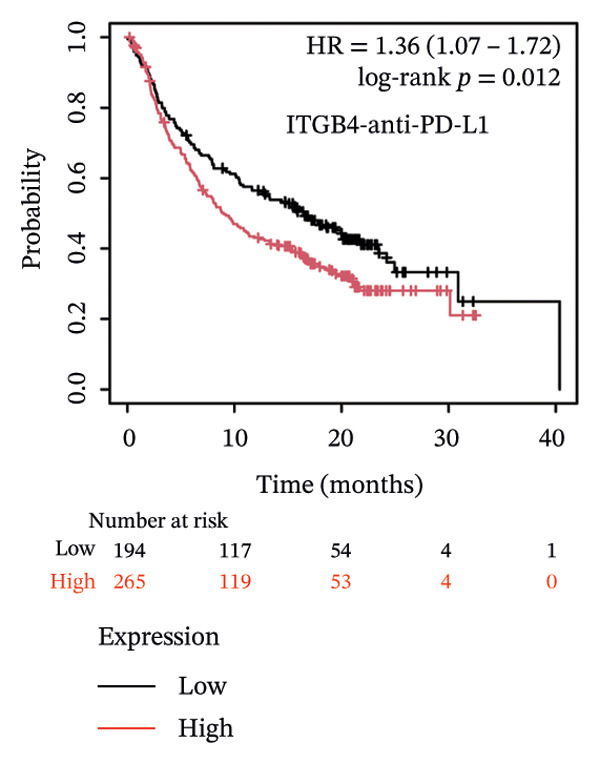
(f)

**Figure:**
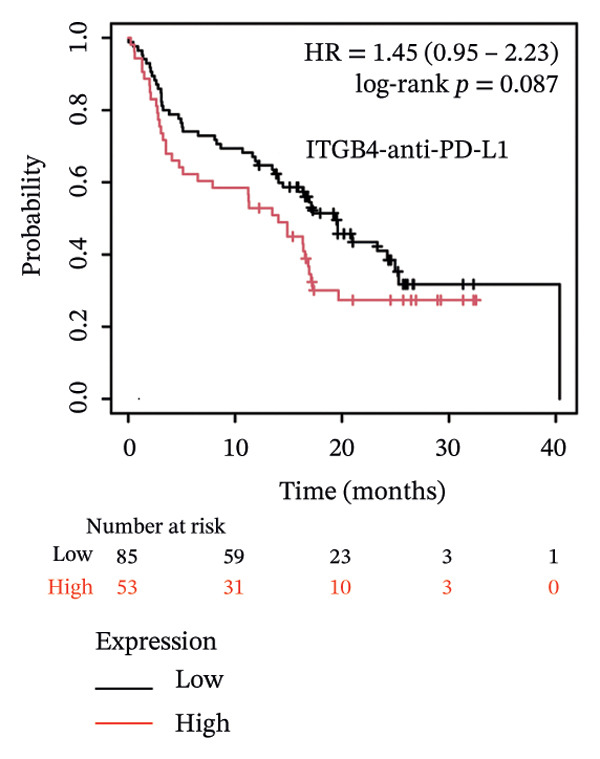
(g)

**Figure:**
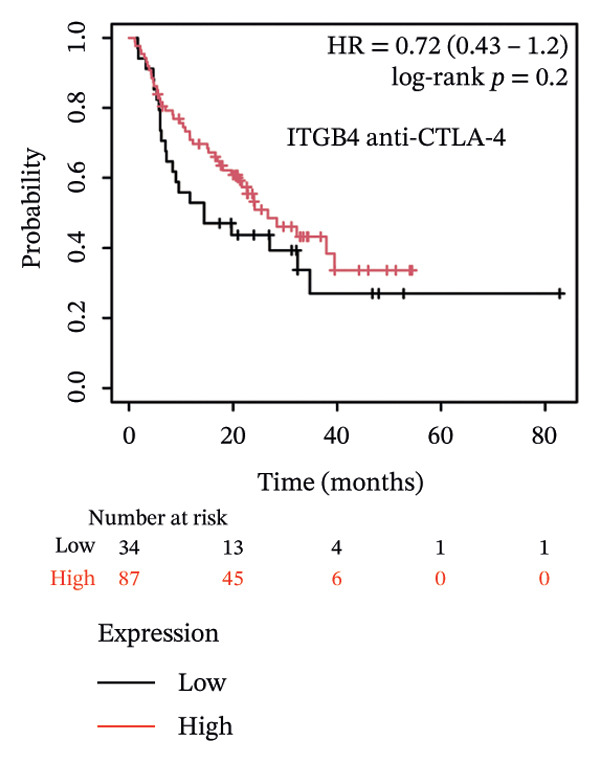
(h)

**Figure:**
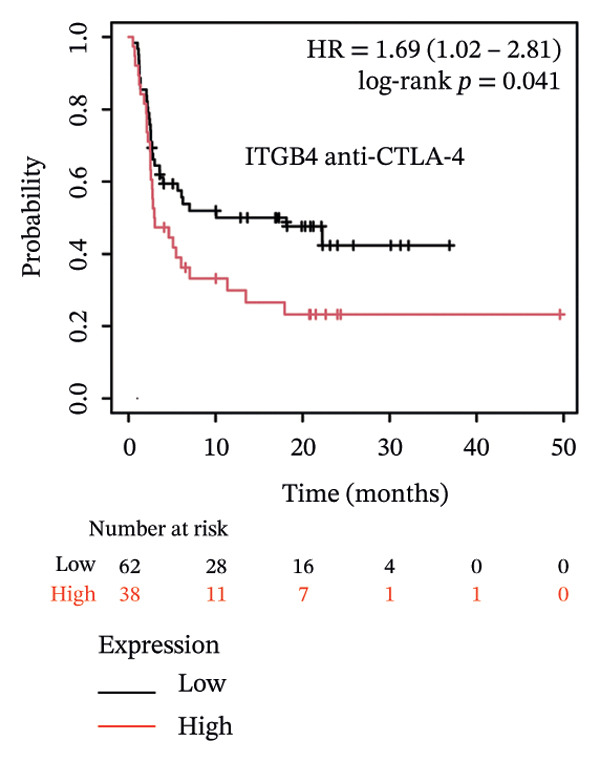
(i)

Given these alterations, we assessed the clinical impact of ITGB4 expression on response to immune checkpoint inhibitors. In cohorts of patients treated with PD‐1 inhibitors, high ITGB4 expression was associated with markedly worse outcomes. ITGB4‐high patients had significantly shorter OS (median OS: 13.32 vs. 25.3 months; HR = 1.62, ^95%^CI:1.22–2.17, *p* = 0.0009; Figure 4(d)) and PFS (median PFS: 2.8 vs. 11.2 months; HR = 2.06, ^95%^CI: 1.59–2.67, *p* = 2.3 × 10^−8^; Figure 4(e)). A similar, though less pronounced, disadvantage was observed in patients receiving PD‐L1 inhibitors, where high ITGB4 predicted inferior OS (median OS: 9.03 vs. 16.46 months; HR = 1.36, *p* = 0.012; Figure 4(f)) but not significantly worse PFS (HR = 1.45, *p* = 0.087; Figure 4(g)). Interestingly, the effect was target‐dependent in patients undergoing CTLA‐4 blockade. High ITGB4 expression was associated with significantly worse PFS (median PFS: 3.3 vs. 18.13 months; HR = 1.69, *p* = 0.041; Figure 4(i)), despite showing no significant association with OS (HR = 0.72, *p* = 0.2; Figure 4(h)).

Collectively, these findings position ITGB4 as a key modulator of the tumor immune contexture and a potent predictor of resistance to checkpoint blockade, with its prognostic value contingent upon the specific therapeutic target.

### 3.5. The ESR1/miR‐342‐5p/UBE2E3 Axis as a Potential Regulator of ITGB4 Protein Stability

We identified UBE2E3 among the overlapping genes between ITGB4 coexpressed genes and genes altered after ITGB4 knockdown (GSE11506) (Figure 5(a)). UBE2E3 decreased upon ITGB4 knockdown (*p* < 0.001; Figure 5(b)), upregulated in breast tumors at the protein level (CPTAC; *p* < 0.001; Figure 5(c)), and correlated positively with ITGB4 mRNA (*R* = 0.33, *p* < 0.001; Figure 5(d)), suggesting a potential feedback circuit involving ubiquitin conjugation and ITGB4 protein stability. Clinically, high UBE2E3 expression predicted poorer OS in luminal breast cancer (TCGA and KM‐Plotter; Figures 5(e) and 5(f)). ESR1 was inversely correlated with UBE2E3 (*R* = −0.40, *p* = 7.5 × 10^−42^; Figure 5(g)). KEGG enrichment of ITGB4‐associated genes highlighted ECM–receptor interaction, focal adhesion, and PI3K–AKT signaling (Figure 5(m)), consistent with an ITGB4‐centered oncogenic program.

FIGURE 5Potential regulatory axis underlying ITGB4 expression discordance and functional pathways. (a) Venn diagram identifying overlapping genes between ITGB4‐coexpressed genes (TCGA–BRCA) and differentially expressed genes upon ITGB4 knockdown (GSE11506). UBE2E3 is highlighted among the 9 overlapping genes. (b) UBE2E3 expression levels following ITGB4 knockdown in the GSE155605 dataset (^∗^ *p* < 0.001, *t*‐test; error bars represent SEM). (c) UBE2E3 protein levels in breast cancer tumors versus normal tissues from CPTAC data (^∗^ *p* < 0.001, Wilcoxon test). (d) Correlation between UBE2E3 and ITGB4 mRNA expression in the TCGA–BRCA cohort (Spearman’s *R* = 0.33, *p* < 0.0001). (e, f) Kaplan–Meier curves for overall survival in luminal breast cancer patients stratified by UBE2E3 expression in (e) the TCGA cohort (HR = 1.7, *p* = 0.022) and (f) a KM‐Plotter cohort (HR = 1.42, *p* = 0.014). (g) Correlation between UBE2E3 and ESR1 mRNA expression in the TCGA–BRCA cohort (Spearman’s *R* = −0.40, *p* = 7.6 × 10^−41^). (h, i) Kaplan–Meier curves for overall survival in luminal breast cancer patients stratified by hsa‐mir‐342‐5p expression in (h) the METABRIC cohort (HR = 0.64, *p* = 0.021) and (i) the GSE40627 cohort (HR = 0.42, *p* = 0.003). (j–l) Correlation analyses in the TCGA–BRCA cohort between hsa‐mir‐342‐5p expression and (j) ITGB4 mRNA (*R* = −0.18, *p* = 0.002), (k) UBE2E3 mRNA (*R* = −0.40, *p* = 9.87 × 10^–13^), and (l) ESR1 mRNA (*R* = 0.47, *p* = 1.45 × 10^–17^). (m) Bar plot showing significantly enriched KEGG pathways among genes correlated with ITGB4 or altered by its knockdown. The *y*‐axis represents the −log10 (*p* value).

**Figure:**
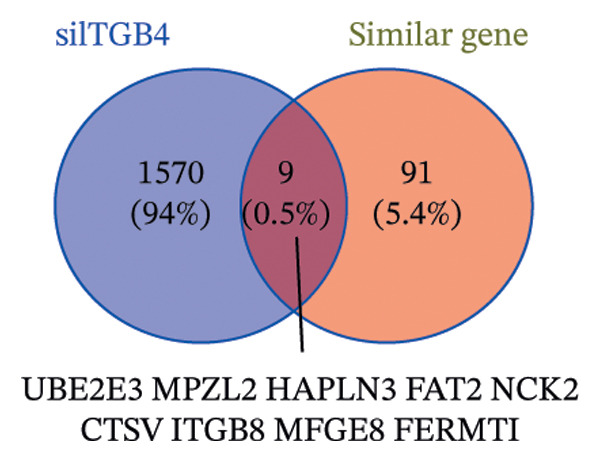
(a)

**Figure:**
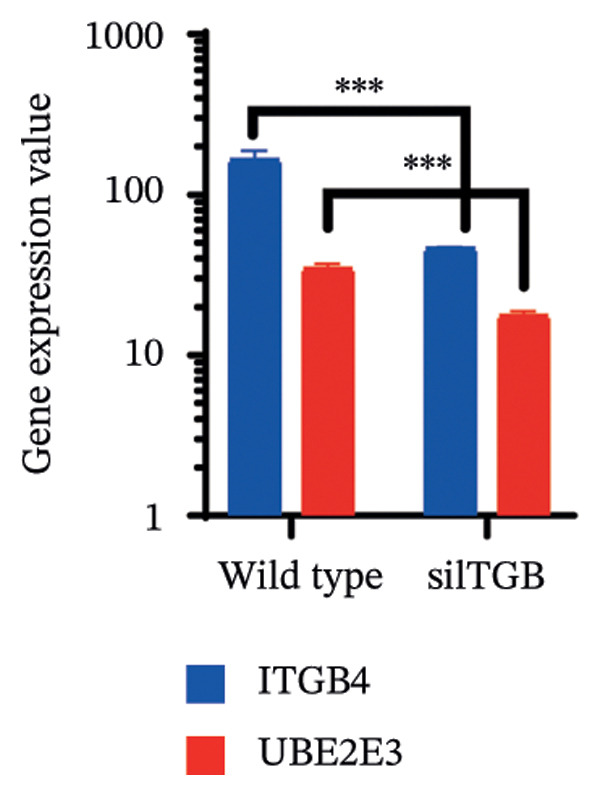
(b)

**Figure:**
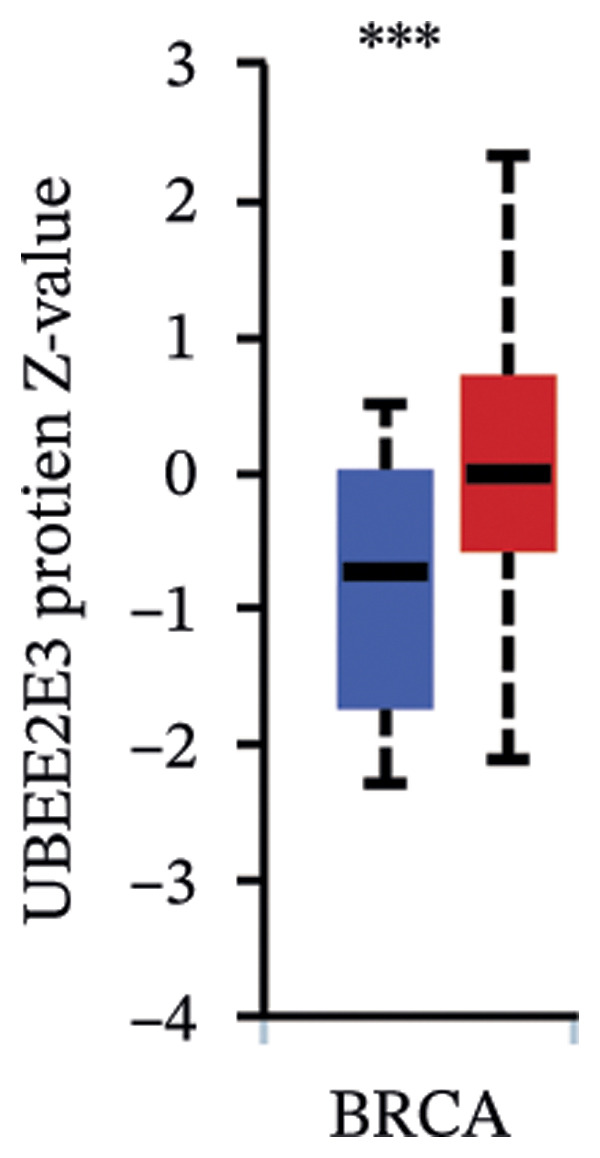
(c)

**Figure:**
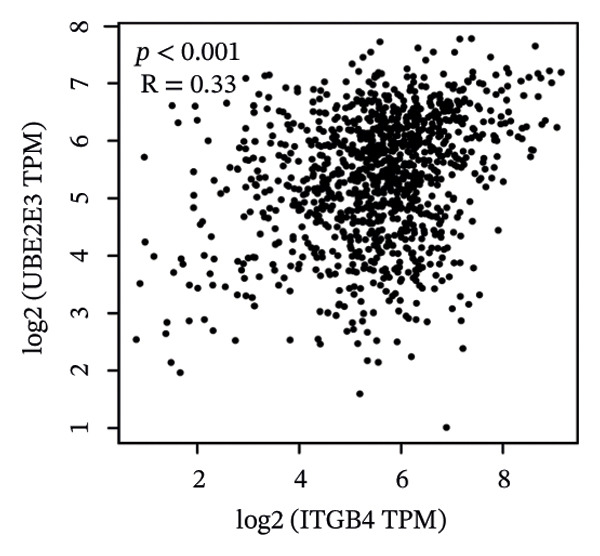
(d)

**Figure:**
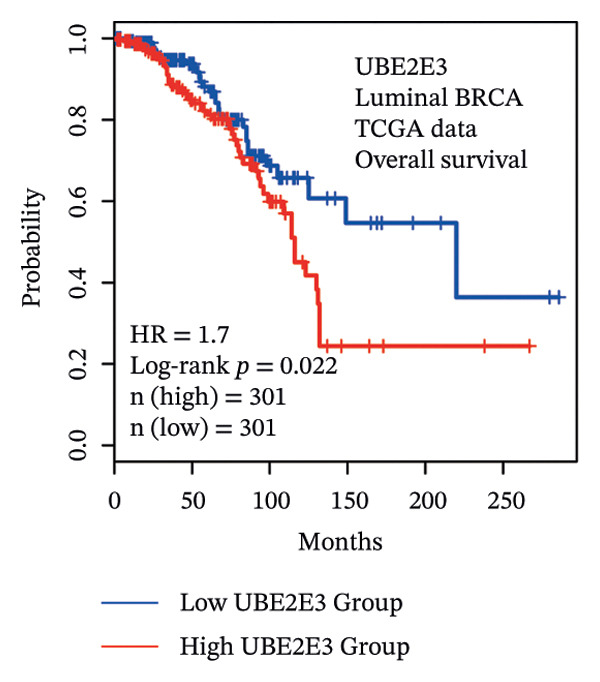
(e)

**Figure:**
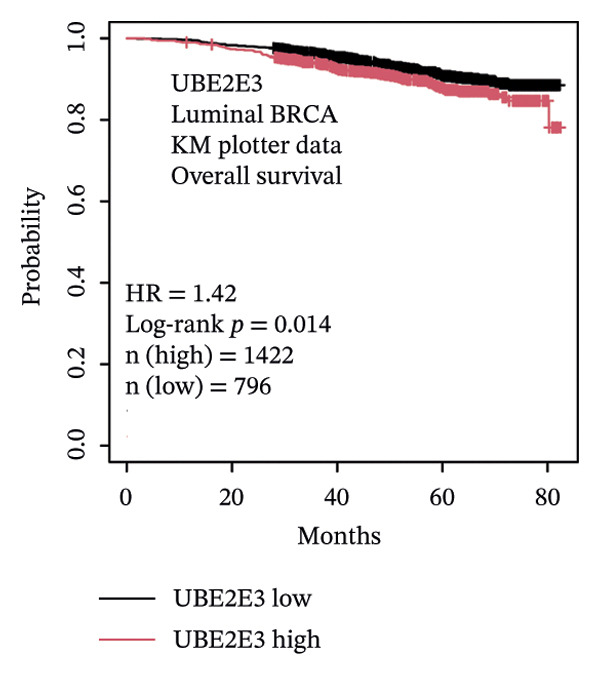
(f)

**Figure:**
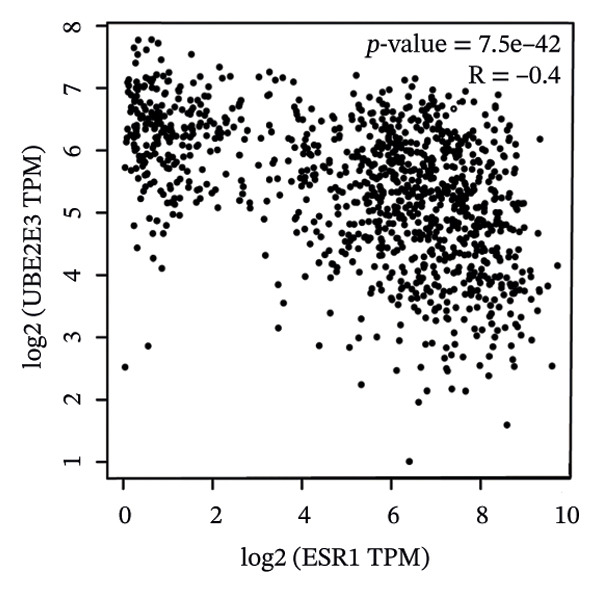
(g)

**Figure:**
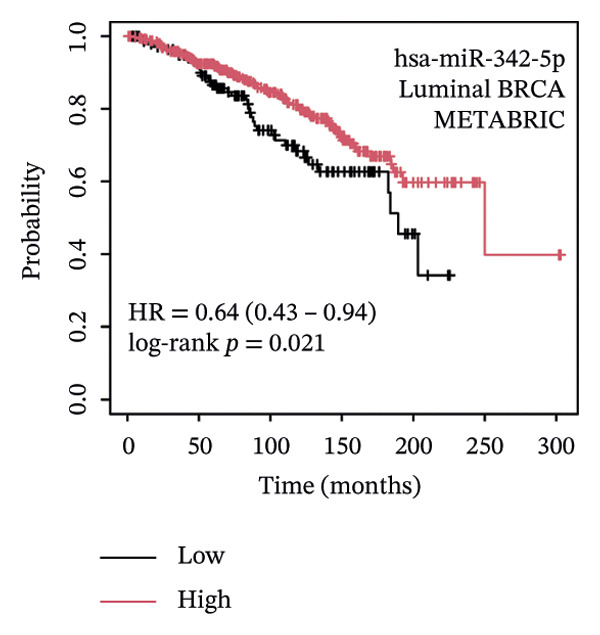
(h)

**Figure:**
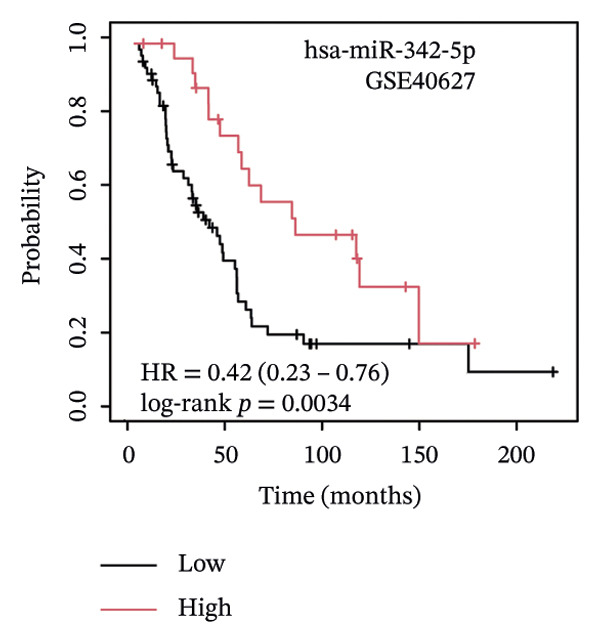
(i)

**Figure:**
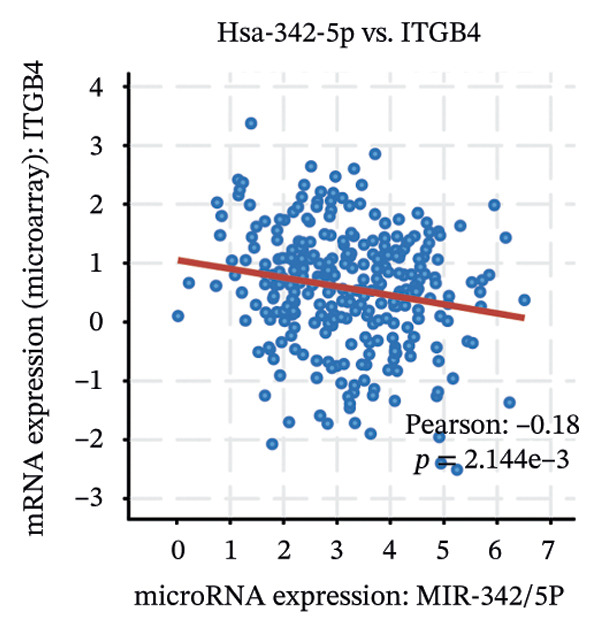
(j)

**Figure:**
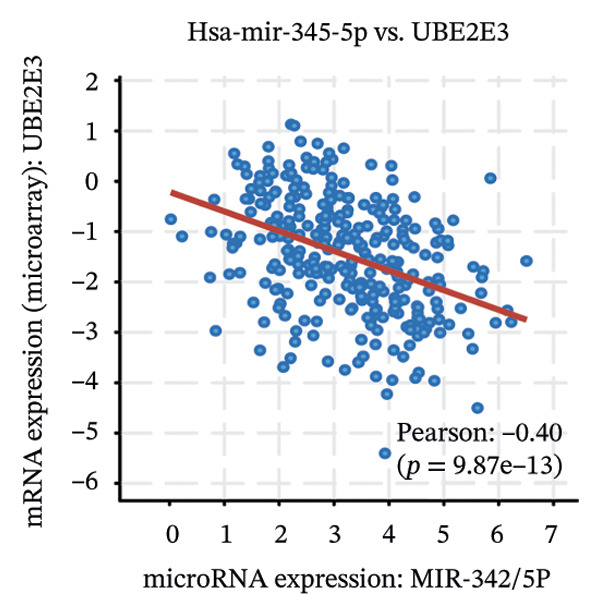
(k)

**Figure:**
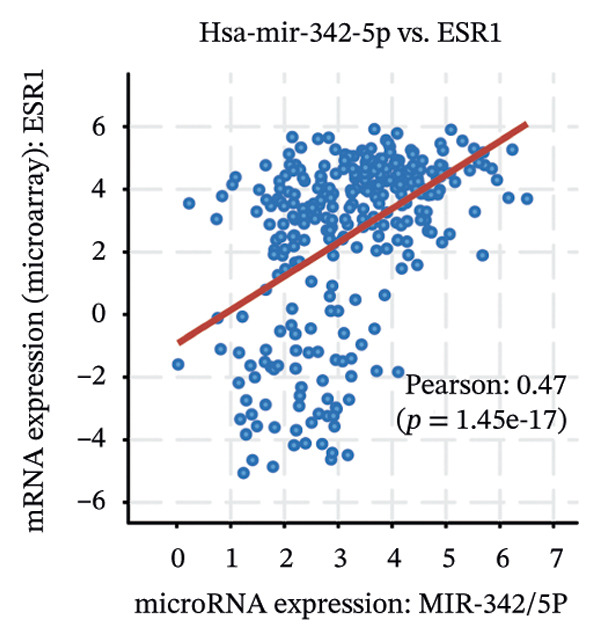
(l)

**Figure:**
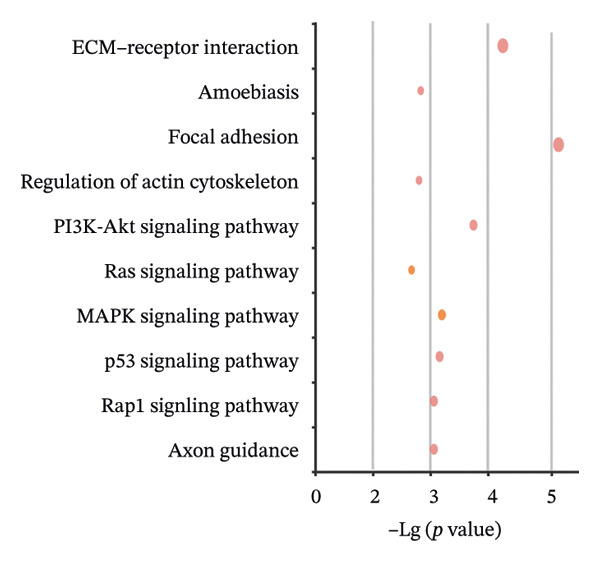
(m)

We interrogated upstream post‐transcriptional and post‐translational regulators. Hsa‐miR‐342‐5p was predicted to target ITGB4 and showed a modest inverse correlation with ITGB4 mRNA in TCGA–BRCA (*R* = −0.18, *p* = 0.002; Figure 5(j)). Consistently, higher miR‐342‐5p expression was associated with improved OS in luminal breast cancer across independent cohorts (METABRIC and GSE40627; Figures 5(h), 5(i)). miR‐342‐5p was positively correlated with ESR1 (*R* = 0.47, *p* = 1.45 × 10^−17^; Figure 5(l)) and was inversely correlated with UBE2E3 (*R* = −0.40, *p* = 9.87 × 10^−13^; Figure 5(k)), supporting an ESR1/miR‐342‐5p‐linked regulatory context in luminal tumors.

## 4. Discussion

In this study, we performed a comprehensive multiomics analysis, revealing that ITGB4 exhibits significant RNA–protein expression discordance across breast cancer subtypes, with important implications for prognosis prediction and therapeutic response. Our findings establish ITGB4 protein, rather than mRNA, as a clinically actionable biomarker with bimodal predictive value, indicating CDK4/6 inhibitor sensitivity in luminal breast cancer while predicting immunotherapy resistance in TNBC.

The observed discordance between ITGB4 RNA and protein levels highlights a critical consideration in biomarker development. While transcriptomic profiling has dominated precision oncology, accumulating evidence demonstrates that protein abundance is often poorly correlated with mRNA levels due to post‐transcriptional and post‐translational regulation [28]. Our data suggest that ITGB4 protein assessment provides superior prognostic information compared to RNA expression, emphasizing the importance of proteomic biomarkers in clinical decision‐making.

The apparent paradox, where high ITGB4 protein correlates with both improved survival and markers of aggressive disease such as high Ki‐67 index, hints at a complex, context‐dependent biology. Rather than solely driving proliferation, high ITGB4 protein may signify a cancer cell state that is more differentiated or structurally integrated within the tumor microenvironment [29, 30]. This is supported by the strong correlation between ITGB4 protein levels and increased tumor–stromal connectivity. We postulate that tumors with high ITGB4 protein may exhibit a greater dependence on adherent, integrin‐mediated survival signals, which could render them more vulnerable to certain targeted therapies (like CDK4/6 inhibitors) and less prone to the dissociative, metastatic behavior associated with the worst outcomes [31, 32]. Thus, the favorable prognosis linked to high ITGB4 protein likely reflects a distinct oncogenic pathway addiction rather than mere indolence, a crucial distinction that has profound implications for treatment selection.

Beyond its prognostic value, ITGB4 protein expression exhibits a compelling dual predictive capacity for response to targeted and conventional therapies. Most significantly, we identified ITGB4 as a novel biomarker predictive of sensitivity to CDK4/6 inhibitors [2]. Pharmacogenomic analyses revealed a strong negative correlation between ITGB4 expression and resistance to palbociclib in luminal breast cancer. This computational finding was functionally validated by our *in vitro* experiments, which demonstrated that treatment with the CDK4/6 inhibitor abemaciclib induced a dose‐dependent suppression of ITGB4 protein levels in MCF‐7 cells. This reciprocal relationship suggests that high baseline ITGB4 protein expression may identify a tumor cell population with a heightened functional dependence on the cyclin D‐CDK4/6 axis for proliferation and survival, a state of oncogenic addiction [33]. Consequently, the abemaciclib‐induced downregulation of ITGB4 may represent both a therapeutic response and a mechanism contributing to the efficacy of CDK4/6 inhibition in this molecular context.

Beyond predicting sensitivity to CDK4/6 inhibition, ITGB4 protein also robustly forecasts resistance to immune checkpoint blockade. In patients receiving PD‐1/PD‐L1 inhibitors, high ITGB4 expression correlates with significantly worse survival, underscoring its clinical relevance. Mechanistically, ITGB4 likely contributes to an immunosuppressive tumor microenvironment through multiple pathways. Our analyses show that ITGB4‐high tumors exhibit elevated expression of immune checkpoint ligands PD‐L1/PD‐L2, which may directly inhibit T‐cell function. Furthermore, these tumors display altered immune infiltration, including reduced cytotoxic lymphocytes, consistent with an immune‐excluded or cold phenotype. Beyond these descriptive features, ITGB4 is known to modulate signaling pathways that foster immune evasion, such as TGF‐β activation and extracellular matrix remodeling, which can recruit immunosuppressive cells like myeloid‐derived suppressor cells (MDSCs) [3, 11]. Its contribution to a predictive immunotherapy gene signature in lung adenocarcinoma reinforces this role in a broader oncological context [34]. ITGB4 has also been linked to epithelial–mesenchymal transition (EMT), a process associated with impaired antigen presentation and enhanced secretion of immunosuppressive cytokines. Thus, ITGB4 is not merely a passive biomarker but an active modulator of immune signaling, positioning it as a critical node linking tumor‐intrinsic dependency on CDK4/6 to extrinsic immune evasion. This dual predictive capacity, sensitizing tumors to targeted therapy while conferring resistance to immunotherapy, provides a strong rationale for ITGB4‐guided therapeutic stratification in breast cancer. The stark contrast in prognostic value between ITGB4 RNA (poor survival) and protein (improved survival) underscores the critical importance of post‐transcriptional regulation in determining its functional outcome [35, 36]. This discordance likely explains the conflicting reports on ITGB4’s role in the literature [9, 12]. The observed divergence between ITGB4 transcript and protein levels suggests the existence of a sophisticated, multi‐tiered regulatory network. Our data suggest a plausible ESR1/miR‐342‐5p/UBE2E3 axis underlying this paradox. In ER‐positive luminal breast cancers, the strong inverse correlation between ESR1 and ITGB4 mRNA implies transcriptional suppression, while miR‐342‐5p, whose expression correlates positively with ESR1 and negatively with ITGB4 mRNA, may act as a post‐transcriptional repressor, potentially mediating transcript degradation or translational inhibition [32, 37]. Conversely, in contexts such as TNBC where ITGB4 protein remains elevated, a post‐translational stabilizing mechanism may operate. The significant coexpression and coregulation of ITGB4 with the ubiquitin‐conjugating enzyme UBE2E3, together with reduced UBE2E3 upon ITGB4 knockdown, hint at a feedback loop. Although UBE2E3 is typically linked to protein degradation [38], it may paradoxically stabilize ITGB4 or modulate its turnover in a context‐dependent manner, consistent with nondegradative ubiquitin signaling [39–42]. Thus, ESR1 and miR‐342‐5p likely suppress ITGB4 at the RNA level, whereas UBE2E3 may help maintain its protein stability, collectively governing the context‐dependent duality of ITGB4 in breast cancer. The regulatory role of the proposed ESR1/miR‐342‐5p/UBE2E3 axis requires functional validation. To dissect this mechanism, future studies should entail manipulating miR‐342‐5p to verify its post‐transcriptional control of ITGB4, coupled with UBE2E3 perturbation and cycloheximide chase assays to explicitly evaluate its effects on ITGB4 protein stability. Our findings both contrast with and clarify prior literature on ITGB4 in cancer. While ITGB4 has been frequently reported as an oncogene promoting aggressiveness in carcinomas such as pancreatic and lung cancer [5–8], our study reveals a more nuanced, subtype‐specific role in breast cancer. This discrepancy underscores the danger of extrapolating a gene’s function across tissue types and molecular contexts without proteomic validation, a limitation increasingly recognized in cancer biology [43]. The key innovation of our work lies in systematically resolving the ITGB4 paradox by demonstrating that its clinically actionable signal is encoded at the protein level, a dimension overlooked by transcriptomic studies alone [44, 45]. Furthermore, by integrating large‐scale multiomics data with functional pharmacological validation and clinical cohort analysis, we move beyond correlative associations to establish a robust, bimodal predictive model for therapy response. This integrated approach provides a definitive framework for understanding ITGB4’s context‐dependent duality.

ITGB4 protein expression demonstrates dual predictive value, making it a compelling stratification biomarker for personalized breast cancer therapy. Clinically, its assessment via standardized IHC could directly inform treatment selection: tumors with high ITGB4 expression may derive greater benefit from CDK4/6 inhibitor‐based regimens, especially in luminal subtypes, while likely exhibiting resistance to taxane‐based chemotherapy and immune checkpoint blockade, particularly in TNBC. Integrating ITGB4 status into prospective clinical trials, especially those involving CDK4/6 inhibitors or immunotherapy combination strategies, is essential to validate its utility. Standardized IHC performed on routine biopsy specimens could guide critical treatment decisions [46]. Further efforts should focus on establishing standardized assays and clinically validated cutoff values to enable its routine application.

Several limitations should be acknowledged. Our *in vitro* validation was limited to MCF‐7 cells; future studies should confirm these findings in additional luminal cell lines with varying baseline ITGB4 expression levels and include ITGB4 knockdown/overexpression experiments to establish causality. While our TMA cohort provides preliminary clinical validation, the limited sample size (*n* = 48) restricts comprehensive subgroup analyses, and larger prospective cohorts are needed. The proposed ESR1/miR‐342‐5p/UBE2E3 regulatory axis requires direct functional validation through mechanistic experiments. The immunotherapy response predictions are based on surrogate markers and retrospective cohorts; prospective validation in patients receiving checkpoint inhibitors is essential.

## 5. Conclusion

In conclusion, our integrated multiomics analysis establishes ITGB4 protein as a bimodal biomarker in breast cancer with distinct clinical implications across molecular subtypes. High ITGB4 protein expression predicts a favorable prognosis and CDK4/6 inhibitor sensitivity in luminal breast cancer, while indicating immunotherapy resistance in TNBC. These findings support the clinical utility of IHC‐based ITGB4 assessment for personalized treatment selection and highlight the importance of protein‐level biomarkers in precision oncology.

**Nomenclature**
BRCA: Breast cancer
CDK4/6: Cyclin‐dependent kinase 4 and 6
CPTAC: Clinical Proteomic Tumor Analysis Consortium
DFS: Disease‐free survival
ER: Estrogen receptor
FBS: Fetal bovine serum
GEO: Gene Expression Omnibus
GDSC: Genomics of drug sensitivity in cancer
HER2: Human epidermal growth factor receptor 2
HR: Hazard ratio
H‐score: Histochemical score
IHC: Immunohistochemistry
ITGB4: Integrin subunit beta 4
MDSC: Myeloid‐derived suppressor cell
OS: Overall survival
PD‐1: Programmed cell death protein 1
PD‐L1/2: Programmed death‐ligand 1/2
PFS: Progression‐free survival
RFS: Recurrence‐free survival
TCGA: The Cancer Genome Atlas
TNBC: Triple‐negative breast cancer
UBE2E3: Ubiquitin‐conjugating enzyme E2 E3

## Author Contributions

Zhi‐Min Zhu: data curation, formal analysis, software, visualization, and writing–original draft. Lei Hu: visualization and validation. Yan‐Wen Ma: writing–review and editing. Qiong‐Ni Zhu: conceptualization, methodology, supervision, writing–review and editing, validation, and funding acquisition.

## Funding

This work was supported by the Clinical Pharmacy Research Fund Project of the Chinese Medical Association Branch of Clinical Pharmacy (Z‐2021–46‐2101–2023), Ruijin Youth NSCF Cultivation Fund (2025PY112).

## Disclosure

All authors reviewed and approved the final manuscript.

## Ethics Statement

The authors have nothing to report.

## Conflicts of Interest

The authors declare no conflicts of interest.

## Data Availability

The data that support the findings of this study are available from the corresponding author upon reasonable request.
